# Unraveling hidden rules behind the wet-to-dry transition of bubble array by glass-box physics rule learner

**DOI:** 10.1038/s41598-022-07170-y

**Published:** 2022-02-24

**Authors:** In Ho Cho, Sinchul Yeom, Tanmoy Sarkar, Tae-Sik Oh

**Affiliations:** 1 Department of Civil, Construction and Environmental Engineering, Iowa State University, Ames, IA 50011 USA; 2 Department of Physics and Astronomy, University of Tennessee, Knoxville, TN 37996 USA; 3 Department of Chemical Engineering, Auburn University, Auburn, AL 36849 USA

**Keywords:** Applied physics, Computational science

## Abstract

A liquid–gas foam, here called bubble array, is a ubiquitous phenomenon widely observed in daily lives, food, pharmaceutical and cosmetic products, and even bio- and nano-technologies. This intriguing phenomenon has been often studied in a well-controlled environment in laboratories, computations, or analytical models. Still, real-world bubble undergoes complex nonlinear transitions from wet to dry conditions, which are hard to describe by unified rules as a whole. Here, we show that a few early-phase snapshots of bubble array can be learned by a glass-box physics rule learner (GPRL) leading to prediction rules of future bubble array. Unlike the black-box machine learning approach, the glass-box approach seeks to unravel expressive rules of the phenomenon that can evolve. Without known principles, GPRL identifies plausible rules of bubble prediction with an elongated bubble array data that transitions from wet to dry states. Then, the best-so-far GPRL-identified rule is applied to an independent circular bubble array, demonstrating the potential generality of the rule. We explain how GPRL uses the spatio-temporal convolved information of early bubbles to mimic the scientist’s perception of bubble sides, shapes, and inter-bubble influences. This research will help combine foam physics and machine learning to better understand and control bubbles.

## Introduction

Two-dimensional (2D) bubble rafts have been extensively studied since their experimental accessibility provides insights into naturally occurring cellular patterns^1–6^. The bubble assembly evolves over time to minimize the overall surface energy^7–9^. If the surface tension and boundary area solely determine the total energy, any reduction of the boundary area will lower the total energy. The bubble raft will therefore evolve towards a pattern with less boundary area. Given the infinite time, this surface energy minimization will bring the complete elimination of domains, leaving one 2D circular bubble filled with air. The structural evolution requires gas transport from the smaller bubbles to larger bubbles across the liquid films leading to the annihilation of the small bubbles and growth of the big ones^10,11^. For ideal dry 2D foams, von Neumann’s law is well known^12^: $\frac{dA}{dt}={K}_{0}\left(n-6\right)$ where $A$ is the area of a bubble under consideration $; {K}_{0}$ is a rate constant governed by the gas-solution physical chemistry, and $n$ is the number of sides of the bubble. This law states that only bubbles with six sides will be stable, i.e., no area change in a six-sided bubble.

Since the 2D bubble raft was initially conceived as a model for grain growth in metals, the temporal change of average area and area distribution of the bubbles has been the focus of many experimental works^2–6,13,14^. The bubble behavior is often analyzed based on collected bubble array images over time. So far, most of the bubble coarsening experiments and simulations involved a large number of bubbles in probing the statistical behavior^2,15–17^. Only a few reports are available for small 2D bubble clusters^11,18,19^. For the small bubble array, the bubbles at the outer rim tend to have only 5 sides − 4 sides in contact with other bubbles and 1 side in contact with air − while inner bubbles prefer 6 sides as the wet foam dries.

When a bubble raft is trapped between two solid plates, the liquid will gradually evaporate from the peripheral bubbles exposed to air, converting the wet foam to dry foam. The distinction between the wet foam and the dry foam depends on the liquid content in the boundaries and vertices. Wet foams, in which liquid takes up 10% of the total volume, are widespread in food, cosmetics, fire extinguishers, and construction materials^6^. As the liquid content decrease, the average number of neighbors will approach six, pointing to the stable shape expected from the von Neumann’s law. Due to this wet to dry transition, it is not straightforward to theoretically simulate the entire lifespan of the bubble raft from the deposition to the aged state. The deterministic modeling based on vertex movement or global energy minimization often assumes a simplified condition of fixed liquid content^20–22^. Going beyond the bubble growth mechanism study, we can apply the 2D bubble raft for materials patterning. Recently, micropost-based foam patterning has been demonstrated to fabricate a transparent conducting silver grid^23^. Through the design of the micropost templates, it was possible to generate foam patterns of the intended size and shape^24–26^. However, the fabrication of the micropost templates is a time and energy-consuming process. It is desired to find a way to print the foam-based grid patterns on a smooth surface without using any template.

**Figure 1:**
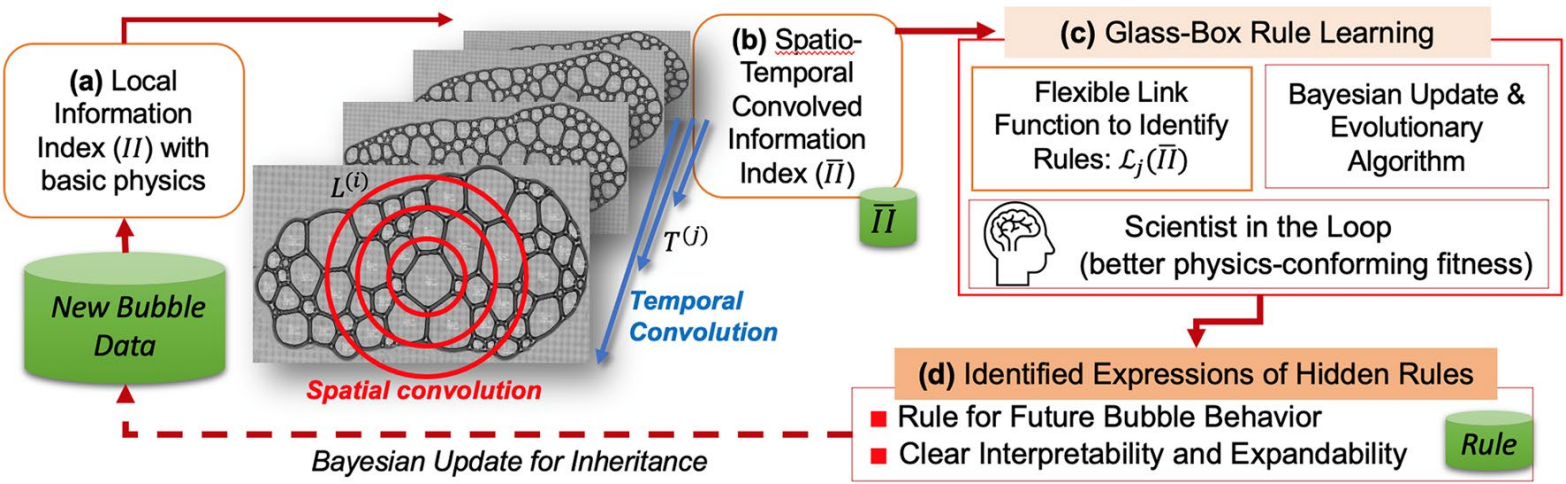
Overall architecture of the glass-box physics rule learner (GPRL) for identifying hidden rules of bubble array (adapted from ^27^): (**a**) Generate local information index with raw bubble data and infused basic physics; (**b**) Externalized information convolution to generate spatio-temporal convolved information index; (**c**) Rule learning core using flexible link functions, a combination of Bayesian update and evolutionary algorithm, and scientist-in-the-loop for infusing scientists’ knowledge into fitness (error) measures; (**d**) Remember the best-so-far expressions of identified rules and smooth knowledge inheritance with new bubble data. Shareable data sets are marked by green cylinders.

Descriptive or governing rules of the aforementioned bubble phenomena in a variety of applications have been often studied in the well-controlled environment in laboratories, computations, or analytical models. Still, real-world bubble undergoes complex nonlinear transitions from wet to dry conditions, which are hard to describe by unified rules as a whole. Here, we show that a few early-phase snapshots of bubble array can be learned by a glass-box physics rule learner (GPRL)^27^ to unravel hidden rules of future bubble array. Figure 1 illustrates the overall architecture of the adopted GPRL framework for unraveling bubble’s wet-to-dry transition rule. As opposed to the so-called “black-box” machine learning approach, the glass-box approach pursues expressive rules of the phenomenon and their transparent interpretability, and smooth evolutions. Without resorting to complex principles of foam physics, GPRL identifies plausible rules of bubble prediction with an elongated bubble array data that transitions from wet to dry states. Then, the best-so-far GPRL-identified rule is applied to an independent circular shape bubble array, demonstrating the potential generality of the rule. We also explain how GPRL uses the spatio-temporal convolved information of early bubbles to help mimic the scientist’s perception of bubble sides, shapes, and inter-bubble influences.

## Results

### Bubble growth image collection

To collect real-time bubble growth images, we deposited 2D bubble rafts on a glass slide using commercial foaming soap (Dial Complete). Another glass slide of the same size covered the bubbles such that only the bubbles at the edges meet air. All inner bubbles touch the two top and bottom glass slides (z-direction) and are surrounded by other bubbles (x-, y-direction). Right before the bubble raft immobilization between the two glass slides, the glass slides were cleaned with water. After washing, the access water was wiped out. The height of the vertical water film was fixed by four same-thickness spacers at the corners of the rectangular glass slides. The 2D bubble raft aged at room temperature. The bubble array images were collected by a USB digital microscope (Mustcam). The transmitted light coming from the bottom was collected from the top for the optical imaging. The time interval between images was fixed at 30 s. For reference, supplementary movies present how the elongated and circular bubble arrays continuously transition from wet to dry states over time.

### Convolved information index by infusing scientists’ knowledge into bubble observations

**Figure 2:**
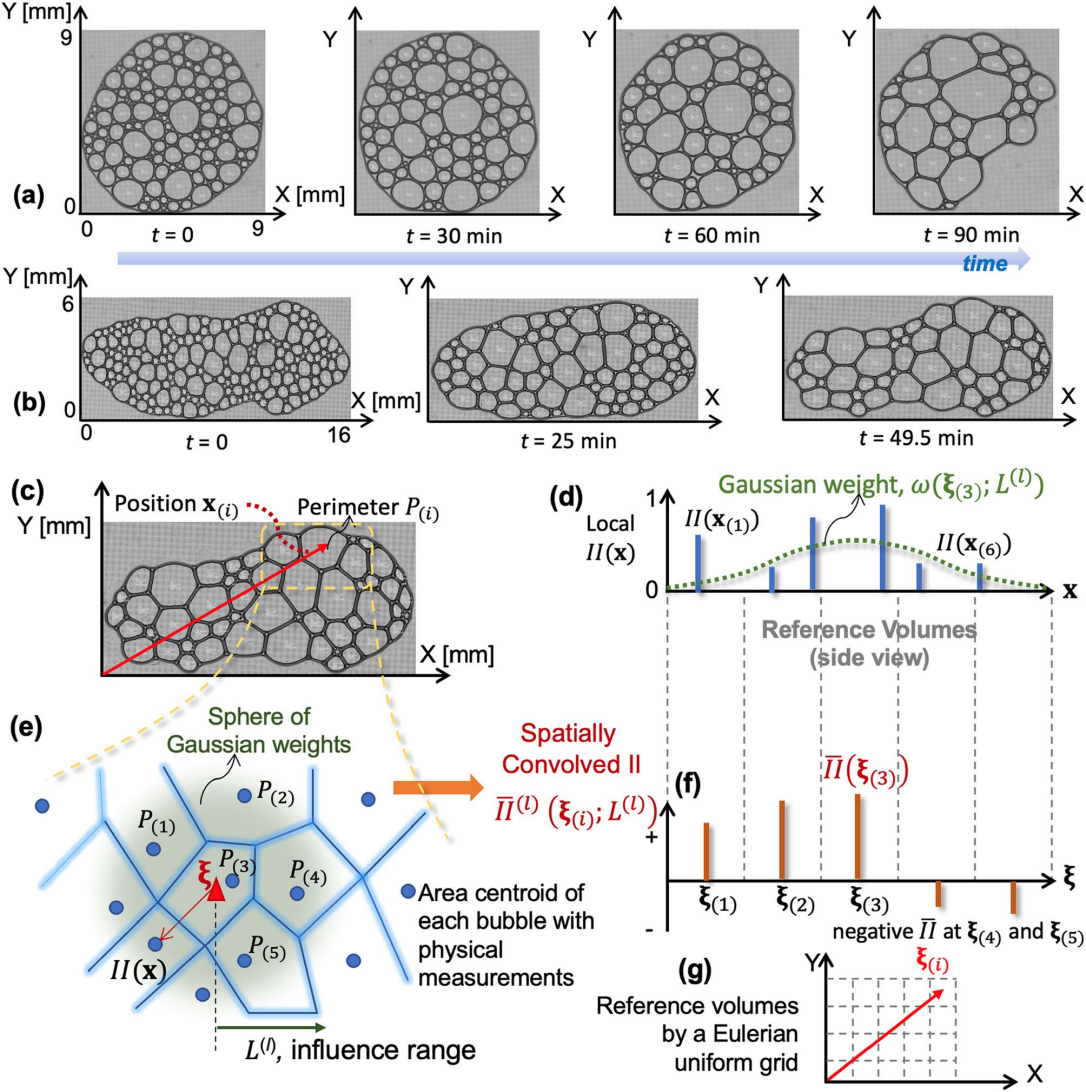
Time-lapsed snapshots of 2D bubble arrays and explanation of the derivation of convolved information index (II): (**a**) Circular bubble array in 9 mm $\times$ 9 mm domain and (**b**) elongated bubble array in 16 mm $\times$ 6 mm domain. Both specimens are placed between two glass plates and the gap is 0.25 mm thick. The time interval between two images is 30 s; (**c**–**g**) Derivation of convolved II and reference volume with a fixed Eulerian uniform grid frame; (**c**) snapshot of a bubble experiment and extraction of perimeters and position vectors; (**d**) local II at the areal centroid of each bubble; (**e**) reference volume (centroid $\upxi$) and spatial convolution; (**f**) spatially convolved II with sign being positive to indicate potential bubble growth and negative to indicate a potential bubble shrinking; (**g**) 2D view of the Eulerian uniform grid system to define reference volumes (i.e., fixed in time unlike the Lagrangian frame).

The number and perimeter sizes of bubbles are constantly evolving by a combination of merging, growing, and slow migrations of centroids. To describe the spatially and temporally evolving bubble growth, we define a fixed Eulerian uniform grid system, the set of reference volumes (Fig. 2c). Unlike the Lagrangian frame, the adopted Eulerian frame is fixed in time to ease the bubble tracking (Fig. 2g). One reference volume may represent multiple bubbles if they are small and reside within the reference volume. The position vector of the centroid of ith reference volume ${V}_{(i)}$ is defined by ${{\varvec{\upxi}}}_{(i)}\in {\mathbb{R}}^{3}$ (Fig. 2e). We introduce local information index (denoted as $II\in {\mathbb{R}}[0,1]$; Fig. 2d) which physically implies (at least indirectly) the mean of surface energy associated with a reference volume: ${\mathbb{E}}\left[{\int }_{{V}_{\left(i\right)}}{\gamma }_{se}dV\right]\simeq \sum_{\forall {\mathbf{x}}_{\left(j\right)}\in {V}_{\left(i\right)}}{\gamma }_{se}{t}_{th}P\left({\mathbf{x}}_{\left(j\right)}\right)$,

1 $$II\left({{\varvec{\upxi}}}_{\left(i\right)}\right)=\frac{\sum_{\forall {\mathbf{x}}_{\left(j\right)}\in {V}_{\left(i\right)}}{\gamma }_{se}{t}_{th}P\left({\mathbf{x}}_{\left(j\right)}\right)}{{\gamma }_{se}{t}_{th}P(\Omega )}=\frac{\sum_{\forall {\mathbf{x}}_{\left(j\right)}\in {V}_{\left(i\right)}}P\left({\mathbf{x}}_{\left(j\right)}\right)}{P(\Omega )}$$

where ${t}_{th}$ is the mean bubble thickness (i.e., the fixed distance 0.25 mm between the two plates); ${\gamma }_{se}$ is the soap water surface energy. In reality, ${\gamma }_{se}$ is changing over time since water evaporation changes the concentration of the soap water. However, this temporal change does not impact $II\left({{\varvec{\upxi}}}_{\left(i\right)}\right)$ since it is normalized by the total surface energy of the bubble assembly as in Eq. (1). Still, the bubbles near outer edges could have surface energy that differs from the rest due to water evaporation from the air–liquid contact surface. For simplicity, we assumed that this will not change the evolution of the overall bubble array for this study. Such a spatial ${\gamma }_{se}$ variation in $II\left({{\varvec{\upxi}}}_{\left(i\right)}\right)$ calculation shall be a future extension topic.

$P({\mathbf{x}}_{\left(j\right)}){\equiv P}_{\left(j\right)}$ means the perimeter of *j-*th bubble of which centroid position vector is ${\mathbf{x}}_{\left(j\right)}$, and $P(\Omega )$ is the normalization-purpose perimeter of the total domain $\Omega$ (1,000 $\mathrm{mm}$ is used for all simulations herein). There is no restriction of $P(\Omega )$ as long as it can guarantee the output range, $II\in {\mathbb{R}}[\mathrm{0,1}]$. For instance, the initial total sum of perimeters of all bubbles of the circular and elongated bubble array specimens of this study is about 200 mm and 310 mm, respectively.

Next, we seek to infuse basic physics knowledge that a smaller bubble is likely merged into adjacent larger bubbles. To infuse this knowledge into quantifiable values, we leverage the information integration power of convolution to give rise to the convolved II (denoted by ${\overline{II} }^{(t)}\in {\mathbb{R}}[-\mathrm{1,1}]$; Fig. 2f) at the current time $t$,

2 $${\overline{II} }^{(t)}\left({{\varvec{\upxi}}}_{\left(i\right)};{L}^{\left(l\right)}\right)\equiv {\int }_{\forall {\varvec{\upzeta}}\in\Omega }\omega \left(\left|{\varvec{\upzeta}}-{{\varvec{\upxi}}}_{\left(i\right)}\right|;{L}^{\left(l\right)}\right)II\left({\varvec{\upzeta}}\right){I}_{sign}({\varvec{\upzeta}},{{\varvec{\upxi}}}_{\left(i\right)})d{\varvec{\upzeta}}$$

where $\Omega$ means the total domain and ${\varvec{\upzeta}}$ is the position vectors of the other reference volume while ${{\varvec{\upxi}}}_{\left(i\right)}$ is the position vector of *i*-th reference volume. $\omega$ is the Gaussian weight function with influence range ${L}^{\left(l\right)},\boldsymbol{ }(l=1,\dots ,{n}_{l})$ given by $\omega \left(\left|{\varvec{\upzeta}}-{{\varvec{\upxi}}}_{\left(i\right)}\right|;{L}^{\left(l\right)}\right)={\left({L}^{\left(l\right)}\sqrt{2\pi }\right)}^{-3}\mathrm{exp}\left(-\frac{{\left|{\varvec{\upzeta}}-{{\varvec{\upxi}}}_{\left(i\right)}\right|}^{2}}{2{(L}^{\left(l\right)}{)}^{2}}\right)$

${I}_{sign}({\varvec{\upzeta}},{{\varvec{\upxi}}}_{\left(i\right)})$ is the indication function meaning relative difference between two physics quantities (here, perimeters) ${I}_{sign}\left({\varvec{\upzeta}},{{\varvec{\upxi}}}_{\left(i\right)}\right)=1$ if $P\left({\varvec{\upzeta}}\right)\le P\left({{\varvec{\upxi}}}_{\left(i\right)}\right)$ while ${I}_{sign}\left({\varvec{\upzeta}},{{\varvec{\upxi}}}_{\left(i\right)}\right)=-1$ if $P\left({\varvec{\upzeta}}\right)>P\left({{\varvec{\upxi}}}_{\left(i\right)}\right)$. Physically, ${I}_{sign}$ quantifies bubbles’ competition to grow or shrink in view of observational knowledge that the larger surface energy of a bubble, the more likely it tends to grow by absorbing small ones nearby. The adopted Gaussian function’s role is to give a proximity-proportionate weights, and there is ample room to use another weighting function such as a bell-shape or a simple linear weight function. This study prefers the Gaussian function due to its mathematical smoothness and well-proven performance in integrating physical quantities in computational mechanics or nano physics^27,29,31^.

So far, the convolved II can incorporate spatial interactions of bubbles. But, our concern is temporal evolution of bubble growth and thus temporal information about bubbles’ expansion and shrinking is also important. Therefore, we conduct temporal convolution with one-dimensional Gaussian weight function over half temporal space – i.e., “half” is for realizing that only past information affects future bubble, not vice versa. We introduce the spatio-temporal convolved II (denoted by ${\overline{II} }_{ST}^{(t)}\in {\mathbb{R}}[-\mathrm{1,1}]$) at the current time $t$ as ${\overline{II} }_{ST}^{(t)}\left({{\varvec{\upxi}}}_{\left(i\right)};{L}^{\left(l\right)},\boldsymbol{ }{T}^{(k)}\right)\equiv {\int }_{{t}_{past}<t}\omega \left(\left|{t}_{past}-t\right|;{T}^{\left(k\right)}\right){\overline{II} }^{\left({t}_{past}\right)}\left({{\varvec{\upxi}}}_{\left(i\right)};{L}^{\left(l\right)}\right)d{t}_{past}$ where temporal influence range ${T}^{\left(k\right)},\boldsymbol{ }(k=1,\dots ,{n}_{T})$ and $\omega \left(\left|{t}_{past}-t\right|;{T}^{\left(k\right)}\right)={\left({T}^{\left(k\right)}\sqrt{2\pi }\right)}^{-1}\mathrm{exp}\left(-\frac{{\left|{t}_{past}-t\right|}^{2}}{2{(T}^{\left(k\right)}{)}^{2}}\right)$.

Supplementary Fig. 1 shows an example plot of convolved spatio-temporal information index by using four different spatial and temporal influence ranges.

### Link functions for the hidden rule of bubble growth rate

The rate of how fast a bubble at a reference volume will grow or shrink over $\Delta t$ is an unknown physical quantity, of which rule is one of the learning targets of GPRL. In essence, we are not aware of the ground-truth rule of the bubble growth rate (denoted as ${V}_{G}^{(t)}\left({{\varvec{\upxi}}}_{\left(i\right)}\right)$ meaning bubble growth rate at time $t$ at reference volume centered at ${{\varvec{\upxi}}}_{\left(i\right)}$). GPRL uses additive combination of ${n}_{l}\times {n}_{T}$ link functions, each of which unknown free parameters are denoted by ${{\varvec{\uptheta}}}^{\left(l, k\right)}$:

3 $${V}_{G}^{(t)}\left({{\varvec{\upxi}}}_{\left(i\right)}\right)=\sum_{l=1}^{{n}_{l}}\sum_{k=1}^{{n}_{T}}{\mathcal{L}}^{\left(l,k\right)}\left({\overline{II} }_{ST}^{\left(t\right)}\left({{\varvec{\upxi}}}_{\left(i\right)};{L}^{\left(l\right)},\boldsymbol{ }{T}^{\left(k\right)}\right);{{\varvec{\uptheta}}}^{\left(l, k\right)}\right)$$

From the previous works (e.g.,^14^), the general growth rates appear to follow linear or smooth nonlinear forms, and thus we chose two-parameter exponential form for link functions as

4 $$\begin{aligned} {\mathcal{L}}^{{\left( {l,k} \right)}} \left( {\overline{II}_{ST}^{\left( t \right)} \left( {{{\varvec{\upxi}}}_{\left( i \right)} ;L^{\left( l \right)} ,\user2{ }T^{\left( k \right)} } \right);{{\varvec{\uptheta}}}^{{\left( {l, k} \right)}} } \right) & = \left[ {\exp \left( {a_{p}^{{\left( {l, k} \right)}} \left| {\overline{II}_{ST}^{\left( t \right)} } \right|^{{{b_{p}{\left( {l, k} \right)}} }} } \right) - 1} \right]{\text{H}}\left( {\overline{II}_{ST}^{\left( t \right)} } \right) \\ & \quad - \left[ {\exp \left( {a_{n}^{{\left( {l, k} \right)}} \left| {\overline{II}_{ST}^{\left( t \right)} } \right|^{{{b_{n}{\left( {l, k} \right)}} }} } \right) - 1} \right]{\text{H}}\left( { - \overline{II}_{ST}^{\left( t \right)} } \right) \\ \end{aligned}$$

where ${\text{H}}\left( s \right)$ is the heaviside step function meaning $\mathrm{H}\left(s\right)=1$ if $s\ge 0$ and 0 otherwise, and ${{\varvec{\uptheta}}}^{\left(l, k\right)}=\left\{{a}_{p}^{\left(l, k\right)},{b}_{p}^{\left(l, k\right)},{a}_{n}^{\left(l, k\right)},{b}_{n}^{\left(l, k\right)}\right\}$. As seen in Eq. (4), the LF is a combination of two exponential forms, in the positive and negative regimes, respectively. This allows separate rules for how fast bubbles tend to grow or shrink. They may correlate or behave independently, which may be identified by GPRL. Supplementary Fig. 2 shows the possible shapes of the adopted exponential LF, including convex, concave, linear, or near-constant curve.

### GPRL-identified prediction rules

As a preliminary attempt, a short-term prediction rule uses 4 observed snapshots, each separated by $\Delta t=5$ minutes (i.e., 10 time steps apart), to predict the bubble perimeter distribution at 5 min later. This short-term prediction training is used to confirm the overall performance of the GPRL. In the preliminary investigation, it is found that a simple linear rate (i.e., linear velocity) form appears to successfully describe the short-term prediction rule. The time gap $\Delta t=5$ minutes (i.e., 10 time steps) among the four observed snapshots is the same as the gap between the last observed snapshot and the target. Thus, the short-term prediction rule is simply given as ${\overline{II} }_{ST}^{\left(t+\Delta t\right)}\approx {\overline{II} }_{ST}^{\left(t\right)}+\Delta t\times {V}_{G}^{(t)}\left({{\varvec{\upxi}}}_{\left(i\right)}\right)$. Supplementary Fig. 3 shows the short-term 5-min prediction result generated by the best-so-far rule of GPRL with the elongated bubble array.

Contrarily, the long-term prediction training uses the shorter 4 observed snapshots, i.e., each separated by $\Delta t=2$ minutes (i.e., 4 time steps apart), to predict the bubble perimeter distribution at 30 min later. In the long-term prediction a simple linear rate rule may not hold since the gap $\Delta t=2$ minutes (i.e., 4 time steps) among the four observed snapshots is much smaller than the gap between the last observed snapshot and the target, 30 min (60 time steps). GPRL seeks to learn an additional rule about the impact of the long-term time lapse between the last observed snapshot and the future target by replacing $\Delta t$ with a new LF ${\mathcal{L}}_{LP}$ as

5 $${\overline{II} }_{ST}^{\left({t}_{LP}\right)}\approx {\overline{II} }_{ST}^{\left(t\right)}+{V}_{G}^{\left(t\right)}\left({{\varvec{\upxi}}}_{\left(i\right)}\right){\mathcal{L}}_{LP}\left({t}_{LP};{{\varvec{\uptheta}}}_{LP}\right)$$

where ${t}_{LP}$ is the long-term future target’s global time measured from the onset of the initial observation; the additional unknown link function of long-term prediction rule is given by ${\mathcal{L}}_{LP}\left({t}_{LP};{{\varvec{\uptheta}}}_{LP}\right)=\mathrm{exp}\left({a}_{LP}\times {\left({t}_{LP}/{t}_{total}\right)}^{{b}_{LP}}\right)-1.0.$ Here, ${t}_{total}=600$ time steps (i.e., 5 h) is used for normalization; the additional free parameters ${{\varvec{\uptheta}}}_{LP}=({a}_{LP},\boldsymbol{ }{b}_{LP})$ are added to the global free parameters as $\Theta =\left\{{{\varvec{\uptheta}}}^{\left(l, k\right)}, \left(l,k=\mathrm{1,2}\right); {{\varvec{\uptheta}}}_{LP}\right\}.$ By comparing Eq. (5) to the short-term growth rule ${\overline{II} }_{ST}^{\left(t+\Delta t\right)}\approx {\overline{II} }_{ST}^{\left(t\right)}+\Delta t\times {V}_{G}^{(t)}\left({{\varvec{\upxi}}}_{\left(i\right)}\right)$, we notice that the linear increase term (i.e., $\Delta t{V}_{G}^{(t)})$ is generalized by a nonlinear term (i.e., ${\mathcal{L}}_{LP}{V}_{G}^{\left(t\right)}$). In the short-time period, the bubble growth may be assumed linearly dependent on a time increment $\Delta t$, but for a long-time period it is more reasonable to allow a complex nonlinear growth. This is the physical rationale behind nonlinear exponential form of ${\mathcal{L}}_{LP}$, which can sufficiently span the convex and concave nonlinearties as shown in Supplementary Fig. 2. Supplementary Fig. 4 illustrates the smooth evolution over the long-term training epochs.

Finally, GPRL predicts a future perimeter at the ith reference volume by ${P}_{GPRL}\left({{\varvec{\upxi}}}_{\left(i\right)}\right)=P(\Omega ){\overline{II} }_{ST}^{\left({t}_{LP}\right)}\left({{\varvec{\upxi}}}_{\left(i\right)}\right)$ where $P(\Omega )$ is the normalized constant in Eq. (1) and ${\overline{II} }_{ST}^{\left({t}_{LP}\right)}$ from Eq. (5). The sum of observed real perimeters at the ith reference volume is ${P}_{Real}\left({{\varvec{\upxi}}}_{\left(i\right)}\right)=\sum_{\forall {\mathbf{x}}_{\left(j\right)}\in {V}_{\left(i\right)}}P\left({\mathbf{x}}_{\left(j\right)}\right).$ Thus, the mean absolute error (MAE) is defined as $\mathrm{MAE}={n}_{\Omega }^{-1}\sum_{\forall {{\varvec{\upxi}}}_{\left(i\right)}\in\Omega }\left|{P}_{GPRL}\left({{\varvec{\upxi}}}_{\left(i\right)}\right)-{P}_{Real}\left({{\varvec{\upxi}}}_{\left(i\right)}\right)\right|/{P}_{Real}\left({{\varvec{\upxi}}}_{\left(i\right)}\right)$ where ${n}_{\Omega }$ is the number of total reference volumes.

**Figure 3:**
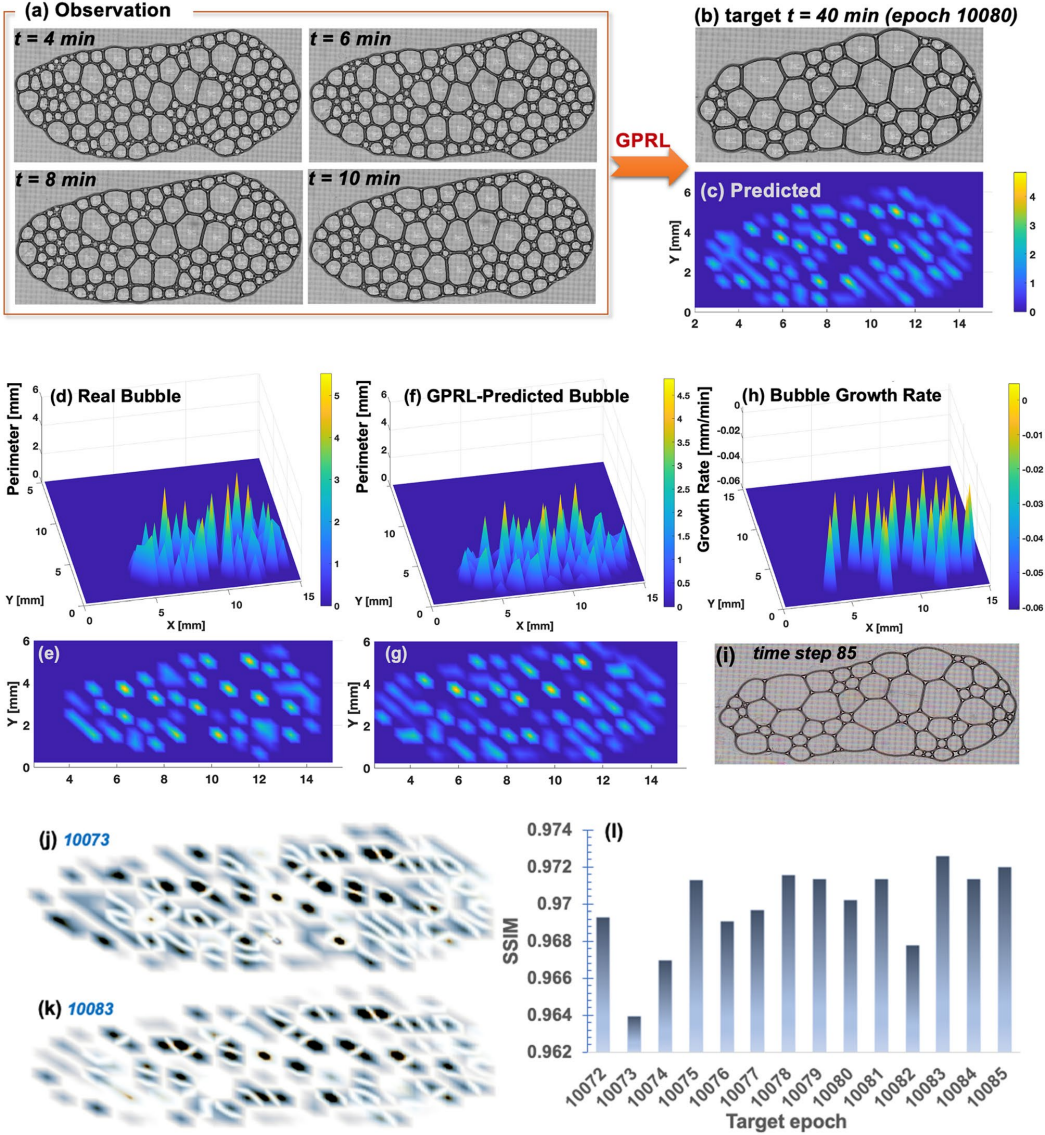
Long-term prediction by the best-so-far rules from GPRL with elongated bubble array: (**a**) shows four real bubble arrays fed into training by GPRL. Only four observed snapshots 2 min apart are needed for training of GPRL; (**b**) Snapshot of the 30 min later target; (**c**) Simulated perimeter distribution of bubble array at the target time step predicted by the best-so-far rule of GPRL (MAE = 0.221856); (**d**) and (**e**) are real bubble arrays from bird’s-eye view and plan view, respectively; (**f**) and (**g**) are simulated bubble arrays (MAE = 0.20493); (**h**) Best-so-far bubble growth rate; (**i**) Snapshot of the real bubble array of the elongated specimen at the time step 85. The GPRL predictions during all training require four observed snapshots 2 min apart to predict bubble 30 min later; (**j**–**k**) Local structural similarity (SSIM) index map of target epoch 10,073 (**j**) and 10,083 (**k**) in which white-colored pixel indicates perfect similarity between real and predicted bubble images whereas black color indicates difference; (**l**) Gradual improvement of global SSIM approaching 1.0 (i.e., identical images) as the Bayesian evolution takes place over 14 target epochs.

Figure 3 summarizes the long-term prediction by the best-so-far rules from GPRL with elongated bubble array. MAE = 0.221856 for the target epoch 10,080 (*t* = 40 min; Fig. 3b) and MAE = 0.20493 for the target epoch 10,085 (Fig. 3f–g). MAE gradually decreases from 0.288474 to 0.20493 for the target epochs 10,072 through 10,085, and the entire MAEs for 14 target epochs are summarized in Supplementary Table 1 where ${\mathbb{E}}(\mathrm{MAE})$, $Var\left(\mathrm{MAE}\right),$ and $Stdev\left(\mathrm{MAE}\right)$ are the mean, variance, and standard deviation of the MAE, respectively, calculated from all organisms (i.e., all alternative rules; here 100,000 organisms) of the best-so-far generation of the Bayesian evolutionary algorithm. All statistics of ${\mathbb{E}}(\mathrm{MAE})$, $Var\left(\mathrm{MAE}\right),$ and $Stdev\left(\mathrm{MAE}\right)$ in Supplementary Table 1 gradually decrease, indicating that GPRL-identified rules gradually evolve and Bayesian update works as desired despite some intermediate fluctuations that are typical due to stochastic nature. The error within 20% ~ 29% is relatively large because the wet-to-dry transition is a complex phenomenon and the training data are small. Still, the result demonstrates the potential of GPRL’s rule learning capability notably with just a few early snapshots and smooth evolution with increasing data. Supplementary Fig. 5 visually illustrates how the best-so-far GPRL rule uses the early 4 snapshopts to predict long-term bubble perimeters.

Since GPRL-identified rules generate images of bubble array, it is informative to compare the image similarity between the real observed image and the GPRL-reproduced image. Indeed, as shown in Fig. 3, it is not straightforward to confirm the GPRL’s performance with bare eyes. To quantitatively answer this question, we adopted the structural similarity index measure (SSIM)^32^. Based on three major characteristics of images—luminance, contrast, and structural, SSIM can quantify the similarity between two images; SSIM = 1 means identical two images (see details in Methods). Figure 3l summarizes the evolution of global SSIM confirming that GPRL gradually improves through the Bayesian evolutionary algorithm over the 14 target epochs. For instance, Fig. 3j shows local SSIM map of target epoch 10,073 with global SSIM of 0.96394 which contrasts with Fig. 3k target epoch 10,083 with SSIM = 0.97136. Albeit slightly, dark black areas in the 10,073 epoch’s SSIM map (Fig. 3j) are replaced by brighter areas (Fig. 3k). A brighter SSIM map means similar two images than a darker SSIM map. This quantitatively confirms that GPRL predictions improve by generating more similar images of bubble arrays as evolution occurs.

**Figure 4:**
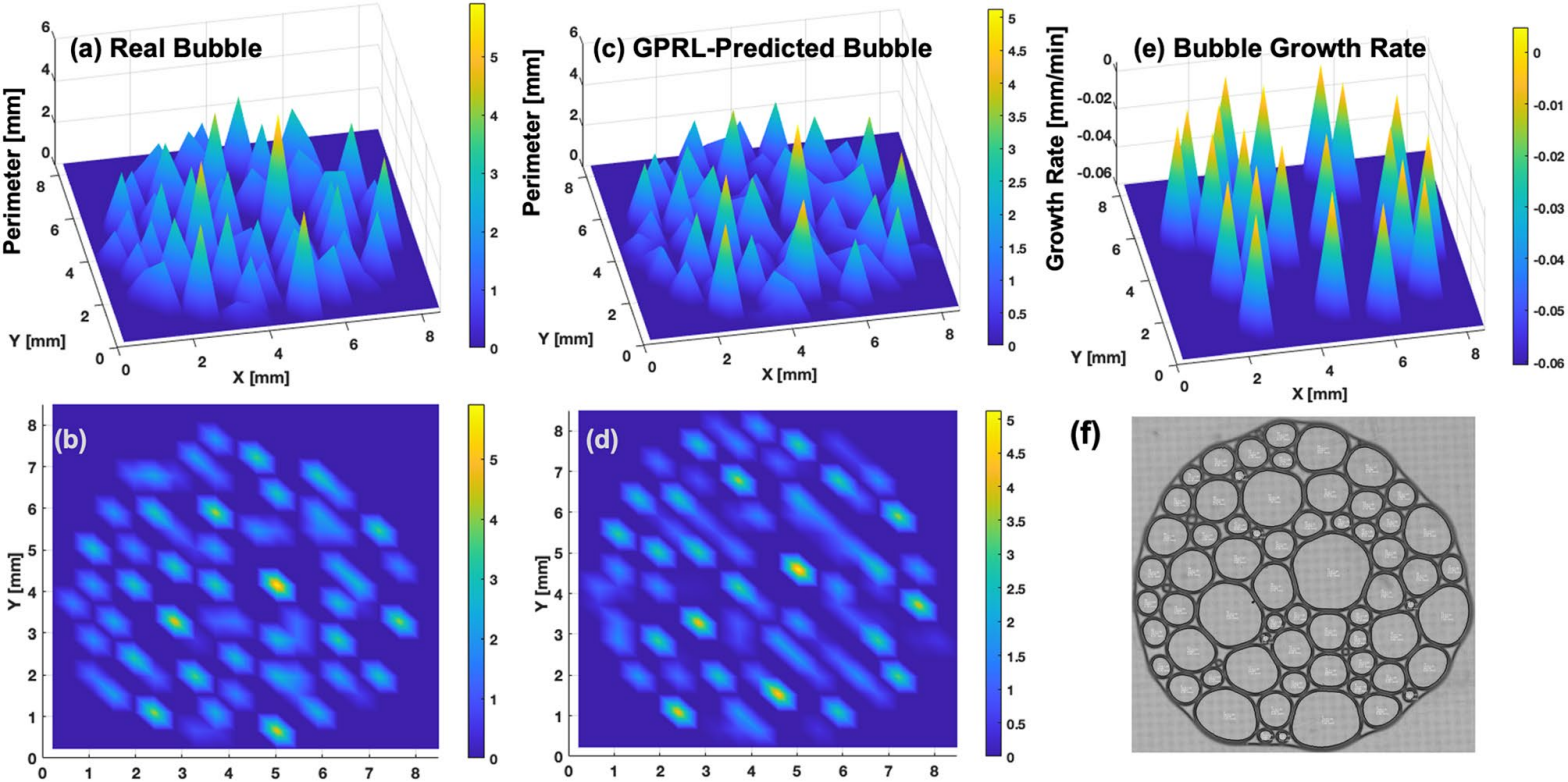
Independent prediction test with the best-so-far long-term prediction rules from GPRL. The GPRL-identified rule is applied to the independent “circular” shape bubble array: (**a**) and (**b**) are real bubble arrays from bird’s-eye view and plan view, respectively; (**c**) and (**d**) are simulated bubble arrays (MAE = 0.0506); (**e**) Best-so-far bubble growth rate; (**f**) Snapshot of the real bubble array of the elongated specimen at the target time step 72 (i.e., *t* = 36 min). Prediction gap = 30 min. The GPRL prediction requires four observed snapshots at *t* = 0, 2, 4, and 6 min to predict bubble at *t* = 36 min.

To confirm the applicability and potential generality of the rules identified by GPRL, the best-so-far rules obtained from the elongated bubble array are applied to an independent circular bubble array. As summarized in Fig. 4, the best-so-far GPRL rule appears to reasonably predict the 30-min future behavior of circular bubble array. The mean absolute error MAE = 0.0506 is calculated from the best-so-far GPRL predictions and observed perimeters of 900 reference volumes. The relatively small MAE of ~ 5% of the circular bubble array may be attributed to the simple shape and arrange of the circular bubble formation compared to the elongated bubble array (Fig. 3). Supplementary Fig. 7 shows the SSIM map (global SSIM = 0.97802) between the real observed image and the GPRL-predicted image of the circular bubble array, confirming the promising prediction performance of the GPRL-identified rules.

Supplementary Table 3 summarizes the entire steps of the future bubble prediction using the GPRL-identified rule expressions. It is important to note that unlike ML methods, GPRL seeks to unravel “expression” and associated free parameters, not merely outcomes. Also, as explained in Supplementary Table 3, GPRL can obtain expression at each prediction step (like the individual layer of deep learning). Such a layered set of expressions offer GPRL with transparency and modularity, which will help researchers interpret each layer’s disparate physics and rationally replace the previous rule with a better rule. Indeed, via the proposed approach, data-learning, prediction, and improvement can take place in a clear “glass box” not a black box.

## Discussion

The qualitative comparison between the proposed GPRL and the generalized bubble coarsening equation is noteworthy. From the well-proven studies^5,30^, von Neumann’s law has been generalized to cover nonlinear wet bubble behavior as

6 $$\frac{dA}{dt}={K}_{0}\left(1-\frac{2r}{H}+\frac{\pi \sqrt{r \ell }}{H}\right)\left(\left(n-6\right)+\frac{6nCr}{\sqrt{3\pi A}}\right)$$

where ${K}_{0}$ is a rate constant governed by the physical chemistry of the surfactant solution and gas; $r$ is the average radius of curvature of the bottom and top surface Plateau borders; $H$ is the height of the 2D bubble; $\ell$ is the width of thin films between two adjacent bubbles; $n$ is the number of sides of the bubble. $C={{A}^{1/2}{\pi }^{-1/2} n}^{-1}({\sum }_{i}^{n}{\kappa }_{i})$ is the “circularity” of the bubble with ${\kappa }_{i}$ being the curvature of its side *i*. Unlike the original von Neumann’s law, this generalization accounts for bubble’s shape and size as well as complex gas diffusion, thereby capable of well explaining nonlinear bubble coarsening for various cases of $n=6$ and $n\ne 6.$

**Figure 5:**
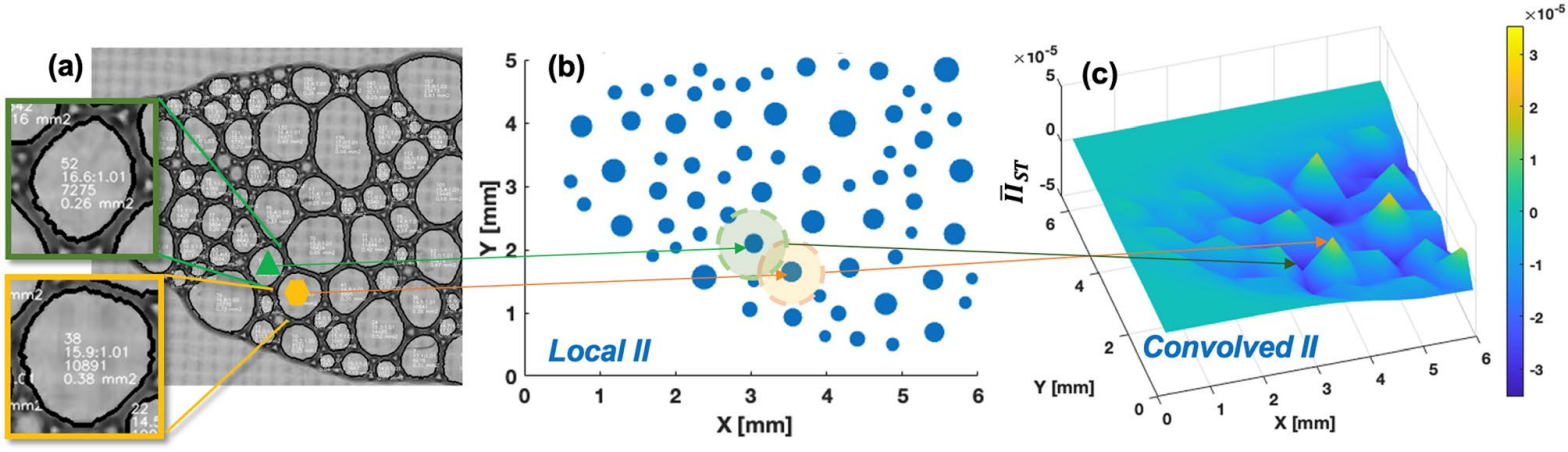
Inter-bubble information extracted by the convolved information index (II): (**a**) Enlarged snapshot at time step 2 of the left part of the elongated bubble array. Yellow hexagon marks the circular shape (i.e., convex bubble $$C>1$$; see a nearly circular shape in inlet) with nearly over six sides ($$n\ge 6)$$ whereas green triangle marks the concave bubble ($$C<1$$; see a nearly rectangular shape in inlet) with less than six sides ($$n<6)$$. White numbers in inlet stand for each bubble’s processed values that mean the bubble index, (pixel perimeter)^2^/(pixel area):(pixel area of the fitted eclipse)/(pixel area), pixel area, and real scaled area, respectively; (**b**) Local II of each bubble by perimeter-proportional scatter plot. Yellow and green shaded regions illustrate the spatial convolution ($$L=0.437$$ mm) centered at the hexagon- and triangle-marked bubbles, respectively; (**c**) GPRL-generated convolved spatio-temporal II distribution with the local maximum near the yellow hexagon and local minimum near the green triangle.

Amongst many terms in Eq. (6), the number of sides $n$ and the circularity $C$ are of central importance and determined by surrounding bubbles. Researchers identified them through prudent observations and statistical investigations. Without measuring these specific terms for each bubble, GPRL appears to capture and quantify such an inter-bubble information (i.e., surrounding bubbles, sides, and thus shapes) via convolved information index (II). As illustrated in Fig. 5, GPRL uses spatial convolution that takes into account surrounding bubbles and indirectly considers the number of sides (*n*) and the shape of the bubble (circularity *C*). In particular, the generalized von Neumann’s law (Eq. (6)) states that a bubble with more than 6 sides and with a circular shape tends to grow. To use Eq. (6), researchers need to measure *n* and calculate *C* from high-precision visual inspections. On contrary, the proposed GPRL conduct a convolution of spatially distributed bubbles (Fig. 5b) to obtain the growth tendancy in a quantified value, i.e., the convolved II (Fig. 5c). Positive values of convolved II (yellow peaks in Fig. 5c) lead to bubble growth whereas negative values (dark blue in Fig. 5c) result in bubble shrinking. As a quantitative example, a seemingly six-sided, circular bubble (marked in yellow box in Fig. 5a) is traced, and the resultant convolved II is confirmed to have a positive peak in Fig. 5c. Another non-circular shape bubble with less sides (*n* < 6) is marked in green box in Fig. 5a and traced, which is confirmed to have a negative convolved II as shown in Fig. 5c. These quantitative tracings assert that without measuring individual terms of Eq. (6), GPRL-generated convolved II appears to distinguish the circular bubble with more than 6 sides from a non-circular bubble with less than 6 sides.

### Nonlinear evolution of bubble growth rate

In summary, the best-so-far 30-min bubble prediction rule identified by GPRL can be re-written by Eq. (3) and Eq. (5) where the best-so-far free parameters ${\Theta }^{*}=\left\{{{\varvec{\uptheta}}}^{*\left(l, k\right)}, \left(l,k=\mathrm{1,2}\right); {a}_{LP}^{*},\boldsymbol{ }{b}_{LP}^{*}\right\}$ and the best-so-far hyper-parameters (i.e., pre-defined parameters associated with GPRL’s global settings) are presented in supplementary Table 2.

**Figure 6:**
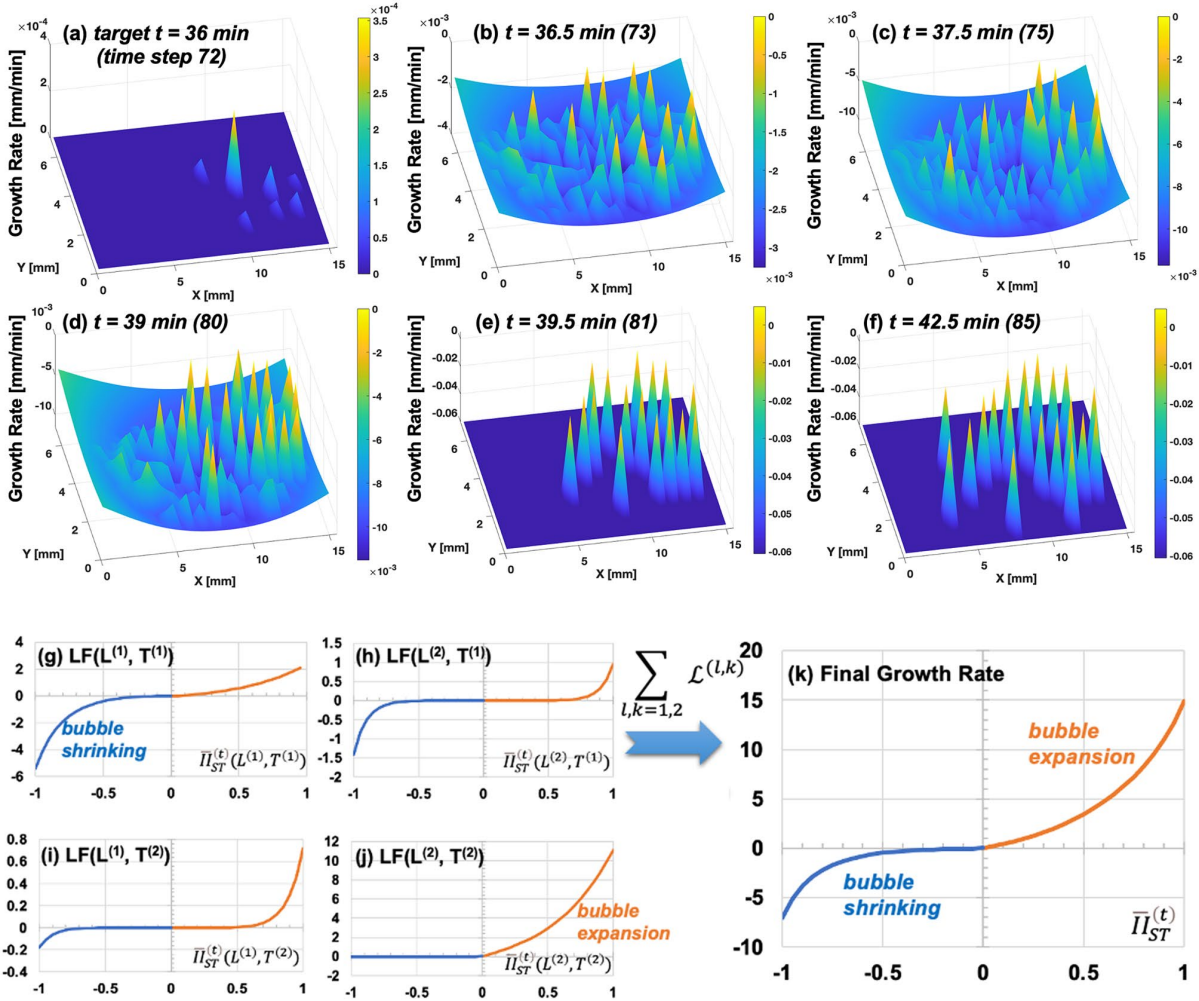
Evolution of the GPRL-identified bubble growth rate at each target 30 min later: (**a**) First GPRL-generated growth rate at target time 36 min using initial four observations (*t* = 0, 2, 4, 6 min) showing weak a few peaks; (**b**) Second GPRL-generated growth rate at target 36.5 min using four observations (*t* = 0.5, 2.5, 4.5, 6.5 min) revealing widespread bubble shrinking rates (rate < 0); (**c**,**d**) GPRL-generated growth rate at target 37.5 and 39 min both resembling the widespread bubble shrinking rates as (**b**); (**e**–**f**) Coexistence of bubble growth (rate > 0) and shrinking (rate < 0) revealed by GPRL. (**g**–**k**) Best-so-far bubble growth rate rules identified by GPRL with the 30-min prediction of elongated bubble array: (**g**–**j**) Decomposed four link functions (LFs) with different spatial and temporal influence ranges. The spatial ranges are [mm] and temporal ranges are [minutes]; (**k**) final bubble growth rate showing asymmetric growth rates in expansion and shrinking regimes.

The original von Neumann law suggests that a dry bubble’s area does not change when the number of bubble side ($n$) is equal to 6 whereas a bubble’s area grows when *n* > 6 and shrinks *n* < 6. As revealed by^14^, wet bubble’s area undergoes a complex growth rate depending upon a variety of factors of size, shape, and film height, thereby suggesting a generalized rule in terms of ${K}_{0}, C\left(t\right), A\left(t\right), H, \ell$ in addition to $n$ (see Eq. 6). Naturally, if a bubble’s state straddles over wet to dry, the underlying bubble growth rate may be complex nonlinear. This paper deals with real-world bubble and its transition from wet to dry states, which imply the growth rates also exhibit a complex nonlinear behavior. Since its onset of learning, GPRL learns and evolves the long-term bubble growth rule with training epochs proceed. Each panel of Fig. 6a–f shows the GPRL-identified rule of the long-term bubble growth at 30 min later. The initially identified rule (Fig. 6a) appears to predict the weak growth of bubble at a few peaks. According to the training error (see supplementary Fig. 5), the first, thus pre-mature, 30-min growth rule appears to have a large error although the genetic algorithm has sufficient searching conditions (100,000 organisms over 30 generations). As GPRL continues to train via the combination of Bayesian update and genetic algorithm, the GPRL-identified rules appear to capture widespread bubble shrinking (negative growth rate; Fig. 6b–d). As time proceeds, GPRL appears to identify a growth rule that allows “coexistence” of bubble expansion (i.e., positive growth rate) and shrinking (Fig. 6e–f). These GPRL-identified rules at later times (e.g., Fig. 6c,f) appear to be reliable, best-so-far rule as supported by the nearly converged learning curve (see the near plateau after the 5th training epoch in supplementary Fig. 5). This result suggests that GPRL may be useful in capturing the nonlinear transition of the complex bubble growth rate evolution and that the bubble growth rates may have a staged-pattern, i.e., widespread shrinking over a certain time period and the coexistence of expanding and shrinking over a later time period. The GPRL’s ability to transparently cover the relatively long process from the wet bubble deposition to the dry state appears to provide a unique predictive capability that will aid the practical foam-based line patterning.

Figures 6g–k shows the summary of the best-so-far bubble growth rate in terms of four decomposed LFs with different spatio-temporal influence ranges (Figs. 6g–j) and the final form of the growth rate (Fig. 6k). Comparing Fig. 6g through Fig. 6j, some interesting tendencies are found. The negative bubble shrinking regime (left half of Fig. 6g) shows a relatively strong tendency to shrinking among the spatiotemporally close bubbles (i.e., ${L}^{\left(1\right)}$ and ${T}^{\left(1\right)}$). This strong bubble shrinking tendency appears to gradually reverse to a strong expansion tendency as the convolved II covers a broader spatial ranges and longer times (Fig. 6j using ${L}^{\left(2\right)}$ and ${T}^{\left(2\right)}$). Ultimately, these four LFs are combined to give rise to a final best-so-far bubble growth rate rule which takes asymmetric nonlinear curves (Fig. 6k). It is noteworthy that all these intriguing tendencies are based on data and the best-performing rules identified by GPRL, not by a pre-defined first principle regarding foam physics. Also, this rule is a best-so-far, not a fixed ground-truth rule. Rather, this rule should evolve with different experimental data and observations. Also, in the future extension, adoption of the Lagrangian frame and inclusion of moving velocity of the reference volume may improve the prediction accuracy, which are feasible in light of the clear interpretability of GPRL. The proposed GPRL approach adds a new dimension to the existing research efforts to help better understand and control complex 2D bubble array during their real-world transition process from wet to dry states.

## Methods

### Reference volumes of the elongated and circular bubble arrays

The elongated bubble array is confined by 15.306 mm 15.306 mm square domain with thickness of 0.25 mm. This global domain is divided by constant thickness reference volumes (0.437 mm $\times$ 0.437 mm) resulting in 1225 (i.e., 35^2^) reference volumes. This long digit value is due to the conversion process of the bubble images from pixels to actual lengths, and there is no specific restriction. Inside a reference volume, there may be more than one small bubble which may be combined to generate one local information index as explained in Fig. 2. The circular bubble array is confined by 13.119 mm $\times$ 13.119 mm square domain with thickness of 0.25 mm. This global domain is divided by constant thickness reference volumes (0.437 mm $\times$ 0.437 mm) resulting in 900 (i.e., 30^2^) reference volumes.

### Convert graphical bubble growth data into ML-friendly data

The microscopic images of bubbles were post-processed by a python script utilizing the OpenCV python library. Outside blank regions of the original images were removed, and the cropped images were enlarged three times to easily identify the edges of the bubbles. The OpenCV’s median blur effect was applied to make edges smoother and the images were converted to black/white images with a threshold value of 127. Then, the contours of the images were identified and non-bubbles were filtered out by our empirical conditions. The centers of mass and arc lengths were calculated from polygons fitted to the bubble contours, and both values were used as positions and perimeters of the bubbles, respectively. The source code of the Python script will be shared upon request to the authors.

### Smooth evolution by a combination of Bayesian update and genetic algorithm

To realize the smooth evolution of the rule-learning of GPRL, this paper adopts the successful combination of the fitness-proportionate probability (FPP) rule of the genetic algorithm^28^ and the Bayesian update scheme as introduced in^29^. The key concept of the smooth evolution of the rule-learning is summarized here and a schematic illustration is shown in supplementary Fig. 4. According to FPP rule, The probability that an organism $s$ (i.e., a candidate for the hidden rules’ free parameters ${\varvec{\Theta}}$) in the current generation is selected as a new parent for next generation is proportional to the fitness score ($\mathcal{F}$), i.e., $p\left({\mathrm{parent}}_{i}| s\right)\propto \mathcal{F}\left(s\right), \left(i=1, 2\right).$ The Bayes theorem inherits the prior knowledge. Given a training (i.e., a certain training epoch of bubble array data set), performing a full genetic algorithm leads to the “best-so-far” generation containing the best organism with the largest fitness score. The prior best generation’s fitness scores are denoted by ${\mathcal{F}}^{*}(s)$, and ${S}^{\mathbf{*}}({\varvec{\Theta}})$ denotes the set of ${\varvec{\Theta}}$ s of the prior best generation. ${\mathcal{F}}^{*}(s)$ is combined with the current fitness scores $\mathcal{F}(s;{S}^{*}\left({\varvec{\Theta}}\right))$ according to the Bayes theorem where $\mathcal{F}(s; {S}^{*}\left({\varvec{\Theta}}\right))$ means the new fitness scores obtained from the new training epoch using the prior best parameter set ${S}^{*}({\varvec{\Theta}})$. With $s$ being a unique realization of ${\varvec{\Theta}}$, $s$ and ${\varvec{\Theta}}$ are interchangeable, and from the FPP rule, $p\left(s\right)\propto \mathcal{F}\left(s\right)$ or equivalently $p\left({\varvec{\Theta}}\right)\propto \mathcal{F}\left(s\right).$ This leads to the Bayesian fitness score of an individual new organism (${\mathcal{F}}_{B}\left(s\right)$) as

7 $${\mathcal{F}}_{B}(s)=\frac{1}{\kappa }\frac{\mathcal{F}(s; {S}^{*}\left({\varvec{\Theta}}\right)){\mathcal{F}}^{*}(s)}{{\sum }_{\forall s}{\mathcal{F}}^{*}(s)}$$

8 $$\kappa ={\sum }_{\forall s}\frac{\mathcal{F}(s; {S}^{*}\left({\varvec{\Theta}}\right)){\mathcal{F}}^{*}(s)}{{\sum }_{\forall s}{\mathcal{F}}^{*}(s)}$$

Then, the probability that an organism $s$ is selected as a new parent for next generation is proportional to the Bayesian fitness scores $p\left({\mathrm{parent}}_{i}| s\right)\propto {\mathcal{F}}_{B}\left(s\right), \left(i=\mathrm{1,2}\right)$. The recorded fitness scores are reused by the Bayesian update scheme to inherit the prior knowledge. In this fashion, all the identified LFs (i.e., ${\varvec{\Theta}}$) can smoothly evolve with new experimental data.

### Structural similarity (SSIM) index

To quantitatively compare the GPRL-generated image of bubble array to the real observed image, SSIM^32^ is adopted by this study. SSIM assesses the visual impact of three image characteristics, i.e., structure, contrast, and luminance, each of which is denoted by $s\left(x,y\right), c\left(x,y\right),$ and $l(x,y)$, respectively; Here, $x$ and $y$ denote two images. In a multiplicative combination of these three terms, SSIM of two images $x$ and $y$ is given by

9 $$SSIM\left(x,y\right)={l}^{\alpha }{c}^{\beta }{s}^{\gamma }$$

10 $$l\left(x,y\right)=\left(2{\mu }_{x}{\mu }_{y}+{C}_{1}\right)/\left({\mu }_{x}^{2}+{\mu }_{y}^{2}+{C}_{1}\right)$$

11 $$c\left(x,y\right)=\left(2{\sigma }_{x}{\sigma }_{y}+{C}_{2}\right)/({\sigma }_{x}^{2}+{\sigma }_{y}^{2}+{C}_{2})$$

12 $$s\left(x,y\right)=\left({\sigma }_{xy}+{C}_{3}\right)/({\sigma }_{x}{\sigma }_{y}+{C}_{3})$$

where ${\mu }_{i}$ is the local mean and ${\sigma }_{i}$ is the standard deviation of image *i*; ${\sigma }_{xy}$ is the cross-covariance for two images $x$ and $y$. For identical images, SSIM becomes 1. This study utilized the built-in function “*ssim*” of Matlab*.*

## Supplementary Information

Supplementary Information 1.

Supplementary Video 1.

Supplementary Video 2.
