# Supplementary material for: Unraveling hidden rules behind the wet-to-dry transition of bubble array by glass-box physics rule learner
Source: Sci Rep. 2022 Feb 24;12:3191. doi: 10.1038/s41598-022-07170-y (PMC8873482; doi:10.1038/s41598-022-07170-y)
Supplement: Supplementary file 1 — Supplementary Information 1. [file 41598_2022_7170_MOESM1_ESM.docx]

**Supplementary Information**

**Supplementary Figures**

**
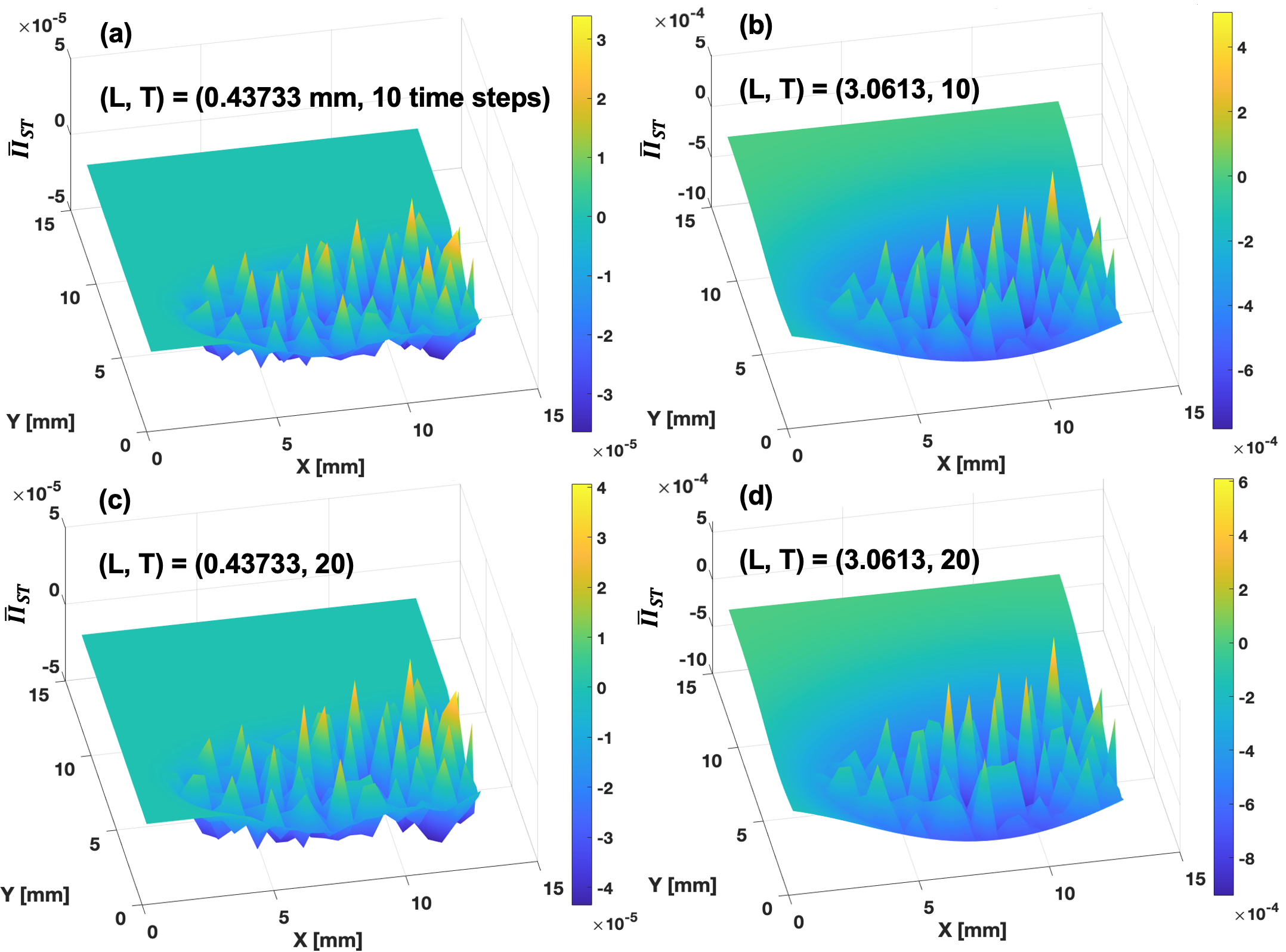
**

**Supplementary Figure 1 Example plots of the convolved spatio-temporal information index (**${\bar{\boldsymbol{II}}}_{\boldsymbol{ST}}^{\boldsymbol{(t)}}\boldsymbol{)}$ **of the elongated bubble**. Each plot is with different spatial and temporal influence ranges, *L* and *T*. The time interval between two consecutive observations is 0.5 minute.


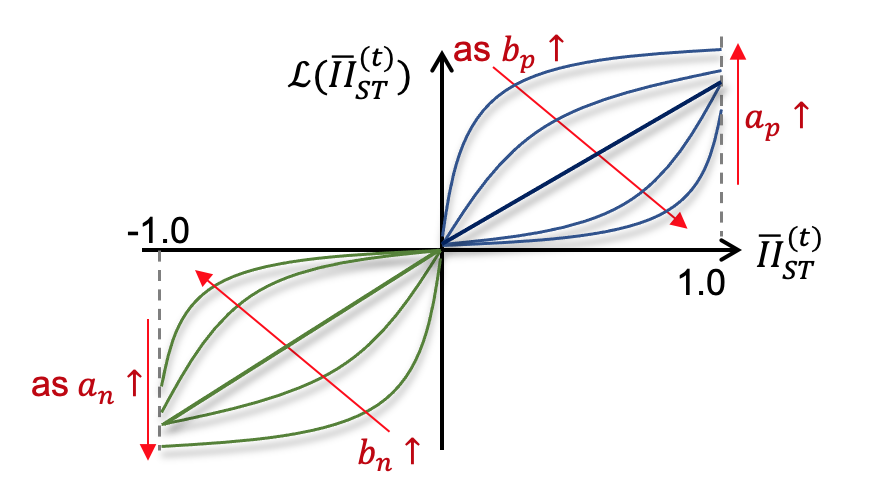


**Supplementary Figure 2 Potential shapes of the bubble growth rate rule to be defined by two exponential form-link functions (LFs).** The regime of the positive spatio-temporal information index ($\bar{II}_{ST}^{\left( t \right)}$ > 0) corresponds to potential bubble growth rate whereas the negative regime ($\bar{II}_{ST}^{\left( t \right)}$ < 0), to bubble shrink.


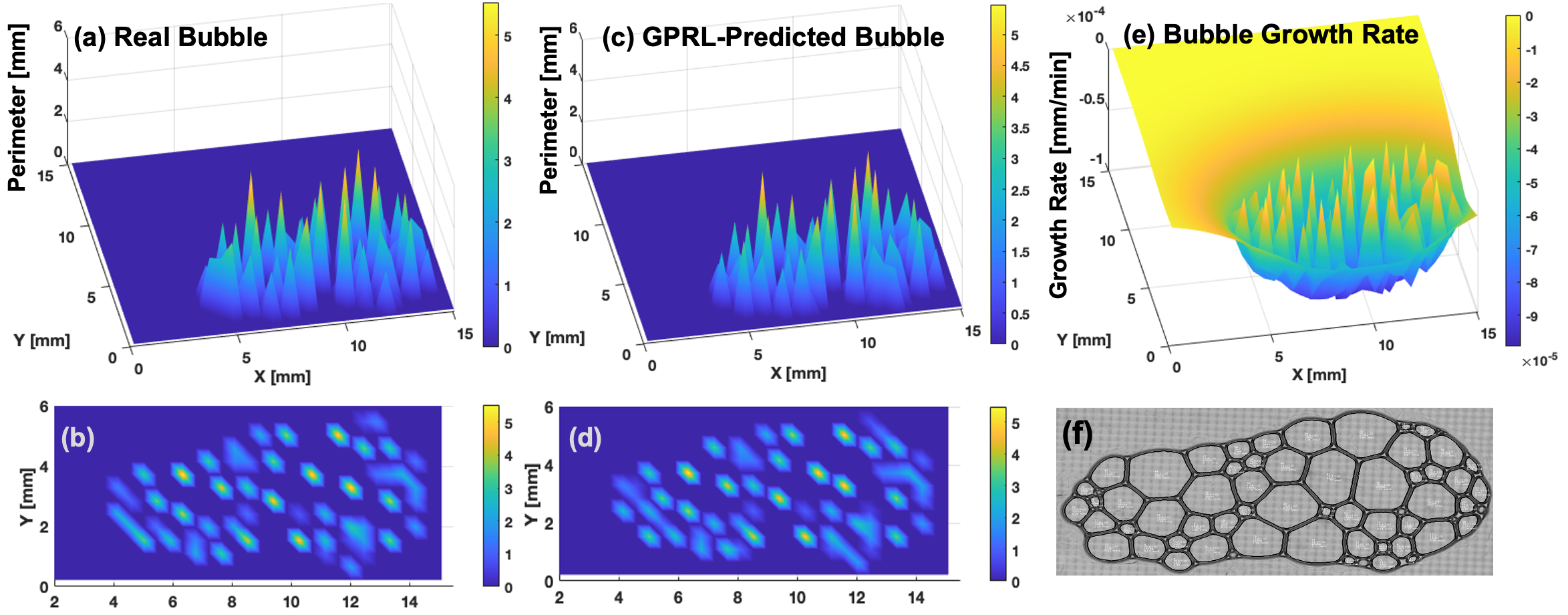


**Supplementary Figure 3 Short-term prediction result generated by the best-so-far rule from GPRL with the elongated bubble array.** (a) and (b) are real bubble arrays from bird’s-eye view and plan view, respectively; (c) and (d) are simulated bubble arrays; (e) Best-so-far bubble growth rate; (f) Snapshot of the real bubble array of the elongated specimen at the target time step 85. Prediction gap = 5 minutes.


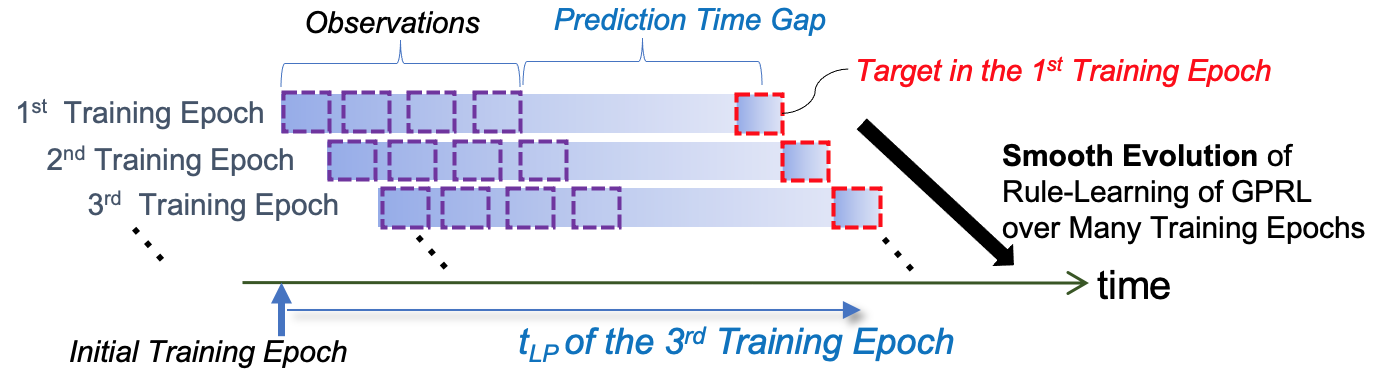


**Supplementary Figure 4 Illustration of the adopted smooth evolution of the GPRL rule-learning.** A combination of Bayesian update and genetic algorithm’s fitness-proportionate probability. The time lapse of “Prediction Time Gap” determines the “short-term” or “long-term” predictions.


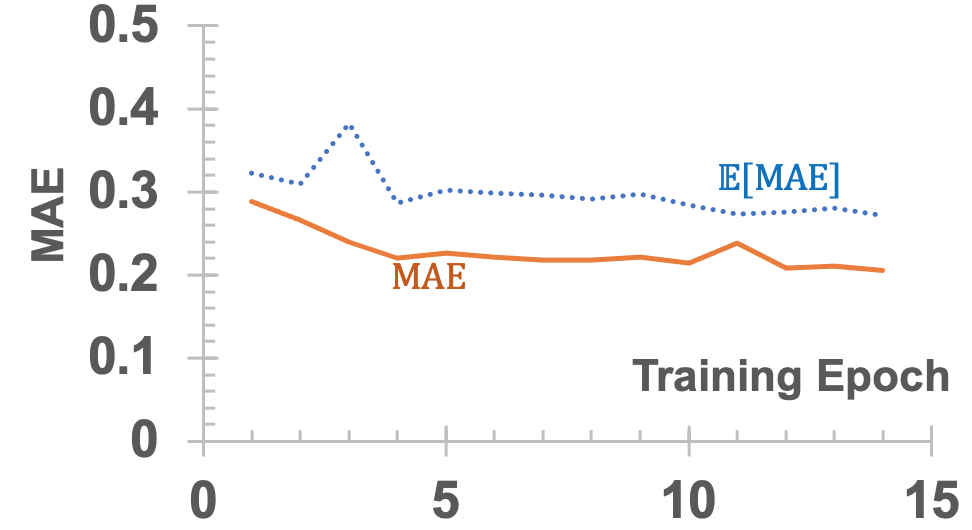


**Supplementary Figure 5 Learning curve of GPRL.** Graph shows the minimum absolute error (orange solid curve) of long-term prediction rule during the training. The mean of absolute errors of all organisms in the best generation is marked by dashed blue curve. Associated variances and standard deviations are presented in supplementary Table 1. The 1^st^ training epoch corresponds to the training case with target time step 72 whereas the 14^th^ training epoch corresponds to target time step 85, each with four observations 30 minutes ahead.

**
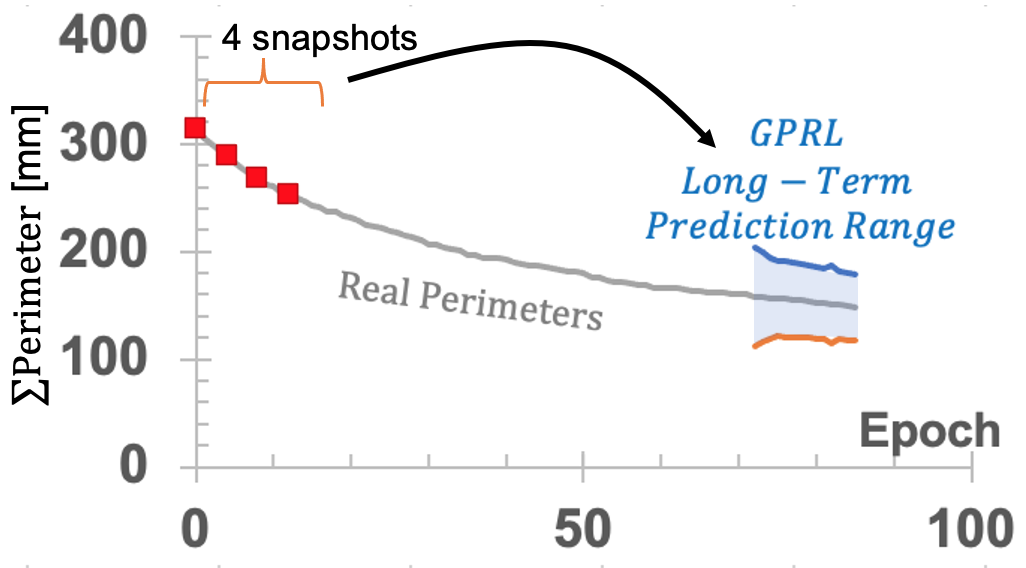
**

**Supplementary Figure 6 Real perimeters and long-term predictions by GPRL-identified rule.** The gray solid line shows the sum of total perimeters of the bubble of the elongated bubble array specimen. The four red points at epoch 0, 4, 8, and 12 are used for the best-so-far GPRL rule to predict perimeters at 30 minutes later. Using the mean absolute error (MAE), the upper and lower bounds of the best-so-far GPRL’s prediction are calculated and marked by shaded zone.

**(b)**

**(c)**

**(a)**


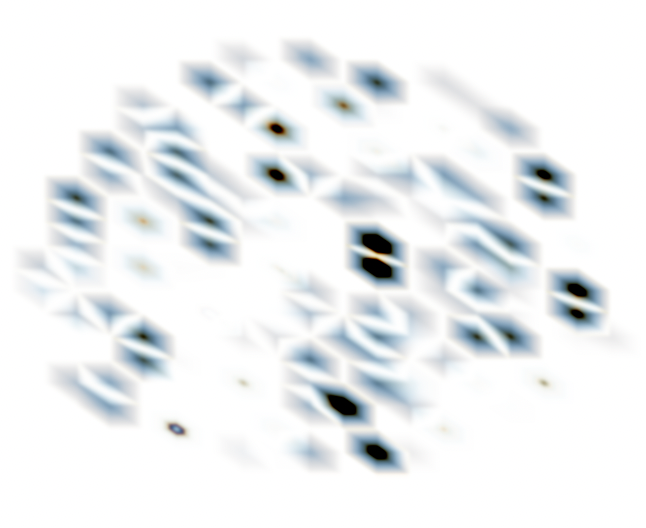

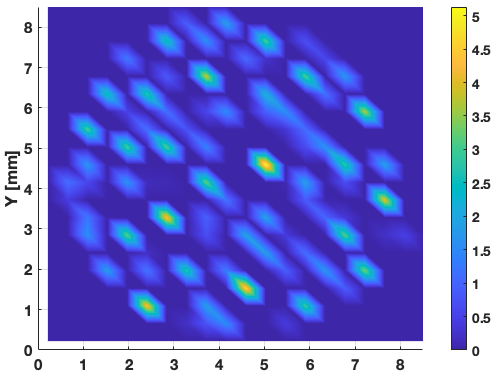

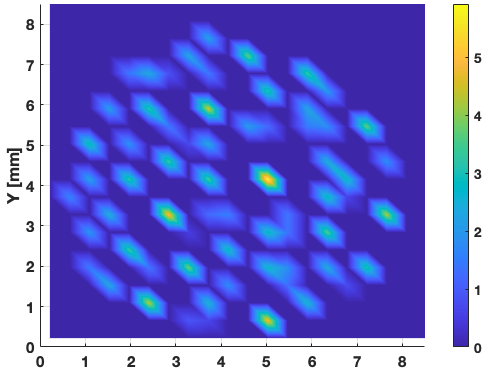


**Supplementary Figure 7 Structural similarity (SSIM) index of GPRL prediction test with the circular bubble array:** (b) the local SSIM map that compares two images of real observed circular bubble array (a) and the GPRL-predicted result (c) (adapted from Fig. 4 of main text). Global SSIM is calculated as 0.97802 (SSIM = 1 means identical two images) which is relatively better than the training results with the elongated bubble array data (Figs. 3(j-l) of main text).

**Supplementary Notes**

**Supplementary Note 1: Learning Curve Results of GPRL**

**Supplementary Table 1.** Summary of the best-so-far long-term prediction rule identified by GPRL with elongated bubble array specimen.


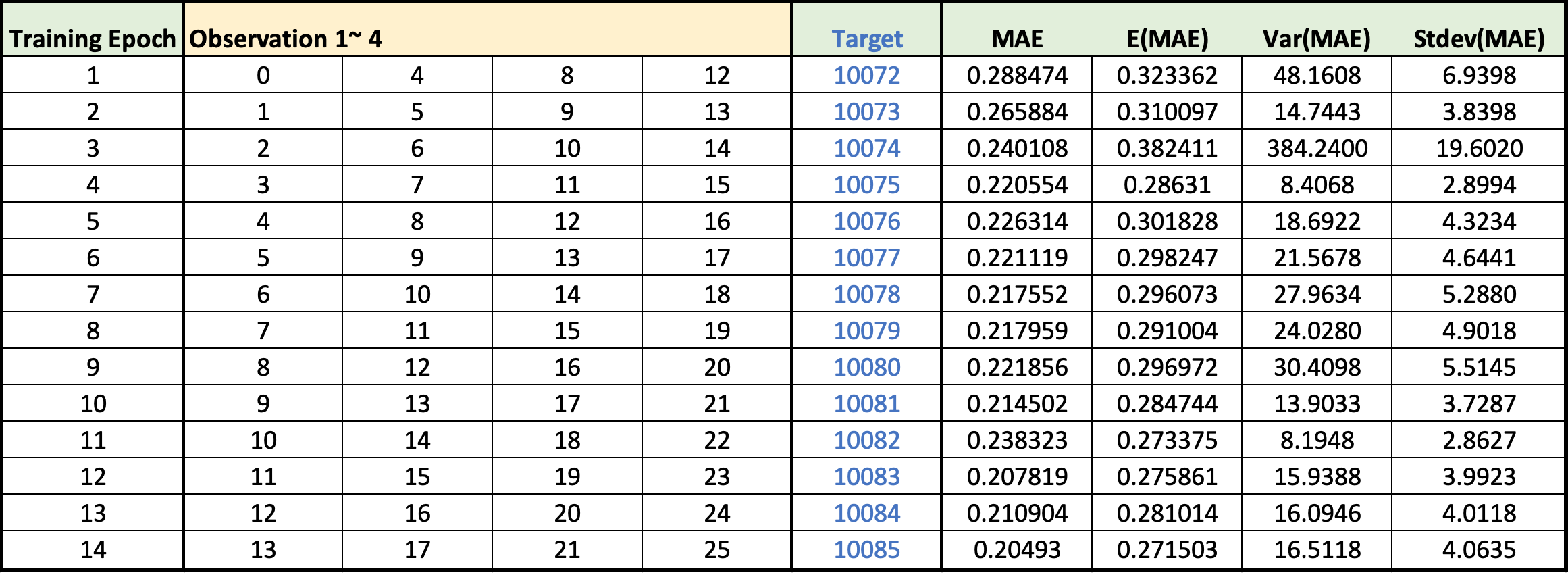


**Supplementary Table 2.** Free-parameters and hyper-parameters of GPRL associated with the identified best-so-far 30-minute bubble prediction rules

| **Free Parameters** | | | | |
| --- | --- | --- | --- | --- |
| Growth rate LFs | $\left( a_{p}^{\left( 1, 1 \right)},b_{p}^{\left( 1, 1 \right)} \right)$ | (1.21176, 1.41176) | $\left( a_{p}^{\left( 1, 2 \right)},b_{p}^{\left( 1, 2 \right)} \right)$ | (0.541176, 7.05882) |
|  | $\left( a_{n}^{\left( 1, 1 \right)},b_{n}^{\left( 1, 1 \right)} \right)$ | (1.85882, 2.47059) | $\left( a_{n}^{\left( 1, 2 \right)},b_{n}^{\left( 1, 2 \right)} \right)$ | (0.164706, 9.29412) |
|  | $\left( a_{p}^{\left( 2, 1 \right)},b_{p}^{\left( 2, 1 \right)} \right)$ | (0.670588, 8.54902) | $\left( a_{p}^{\left( 2, 2 \right)},b_{p}^{\left( 2, 2 \right)} \right)$ | (2.49412, 0.862745) |
|  | $\left( a_{n}^{\left( 2, 1 \right)},b_{n}^{\left( 2, 1 \right)} \right)$ | (0.882353, 7.4902) | $\left( a_{n}^{\left( 2, 2 \right)},b_{n}^{\left( 2, 2 \right)} \right)$ | (0.0588235, 0.0) |
| Long-term time LF | ${(a}_{LP}^{*}\boldsymbol{,}b_{LP}^{*})$ | (0.0117647, 0.0) |  |  |
| **Hyper Parameters** | | | | |
| Influence range | $L^{\left( 1 \right)}$ | 0.4373311 mm | $L^{\left( 2 \right)}$ | 3.0613177 mm |
|  | $T^{\left( 1 \right)}$ | 10 time steps  (5 minutes) | $T^{\left( 2 \right)}$ | 20 time steps  (10 minutes) |
| Search Range | Growth rate LFs | $a_{p \mathrm{or} n}^{\left( i, j \right)}\in[0, 3]$  $b_{p \mathrm{or} n}^{\left( i, j \right)}\in[0, 10]$ | Long-term time LF | $a_{LP}\in[0, 3]$  $b_{LP}\in[0, 10]$ |
| Genetic Algorithm | Organisms | 100,000 | Generations | 30 |
|  | Alleles | 4 | Mutation rate | 0.005 |

**Supplementary Table 3.** Summary of the GPRL-identified expressions of rules for predicting future bubble perimeters. Final prediction expression and all other relevant expressions are given in backward order for illustration purpose.

| **Role of rule** | **Expression of rule identified by GPRL** | **Eq. in text** |
| --- | --- | --- |
| Final prediction | $P_{GPRL}\left( \boldsymbol{\xi}_{\left( i \right)} \right)\boldsymbol{=}P\left( \Omega\right)\bar{II}_{ST}^{\left( t_{LP} \right)}\left( \boldsymbol{\xi}_{\left( i \right)} \right)\boldsymbol{,(}i=1,\ldots, n_{\Omega})$  where $P(\Omega)$ is the normalized constant; $n_{\Omega}$ is the total reference volumes. |  |
| Long-term spatio-temporal II at $t_{LP}$ | $\bar{II}_{ST}^{\left( t_{LP} \right)}\approx\bar{II}_{ST}^{\left( t \right)}+V_{G}^{\left( t \right)}\left( \boldsymbol{\xi}_{\left( i \right)} \right)\mathcal{L}_{LP}\left( t_{LP};\boldsymbol{\theta}_{LP} \right)$  where $\mathcal{L}_{LP}\left( t_{LP};\boldsymbol{\theta}_{LP} \right)=\exp\left( a_{LP}\times\left( t_{LP}/t_{total} \right)^{b_{LP}} \right)-1.0$ | (5) |
| Bubble growth rate | $V_{G}^{(t)}\left( \boldsymbol{\xi}_{\left( i \right)} \right)\boldsymbol{=}\sum_{l=1}^{n_{l}} \sum_{k=1}^{n_{T}} \mathcal{L}^{\left( l,k \right)}\left( \bar{II}_{ST}^{\left( t \right)}\left( \boldsymbol{\xi}_{\left( i \right)}\boldsymbol{;}L^{\left( l \right)}\boldsymbol{,}T^{\left( k \right)} \right);\boldsymbol{\theta}^{\left( l, k \right)} \right)$ | (3) |
| Link function for the bubble growth rate | $\mathcal{L}^{\left( l,k \right)}\left( \bar{II}_{ST}^{\left( t \right)}\left( \boldsymbol{\xi}_{\left( i \right)}\boldsymbol{;}L^{\left( l \right)}\boldsymbol{,}T^{\left( k \right)} \right);\boldsymbol{\theta}^{\left( l, k \right)} \right)$  $=\left[ \exp\left( a_{p}^{\left( l, k \right)}\left\vert\bar{II}_{ST}^{\left( t \right)} \right\vert^{b_{p}^{\left( l, k \right)}} \right)-1 \right]H\left( \bar{II}_{ST}^{\left( t \right)} \right)-\left[ \exp\left( a_{n}^{\left( l, k \right)}\left\vert\bar{II}_{ST}^{\left( t \right)} \right\vert^{b_{n}^{\left( l, k \right)}} \right)-1 \right]H\left( -\bar{II}_{ST}^{\left( t \right)} \right)$ | (4) |
| Spatio-temporal II | $\bar{II}_{ST}^{(t)}\left( \boldsymbol{\xi}_{\left( i \right)}\boldsymbol{;}L^{\left( l \right)}\boldsymbol{,}T^{(k)} \right)\boldsymbol{\equiv}\int_{t_{past}\mathbf{<}t} \omega\left( \left\vert t_{past}\boldsymbol{-}t \right\vert\boldsymbol{;}T^{\left( k \right)} \right)\bar{II}^{\left( t_{past} \right)}\left( \boldsymbol{\xi}_{\left( i \right)}\boldsymbol{;}L^{\left( l \right)} \right)dt_{past}$ |  |
| Convolved II | $\bar{II}^{(t)}\left( \boldsymbol{\xi}_{\left( i \right)}\boldsymbol{;}L^{\left( l \right)} \right)\boldsymbol{\equiv}\int_{\boldsymbol{\forall}\boldsymbol{\zeta\in}\Omega} \omega\left( \left\vert\boldsymbol{\zeta}\boldsymbol{-}\boldsymbol{\xi}_{\left( i \right)} \right\vert\boldsymbol{;}L^{\left( l \right)} \right)II\left( \boldsymbol{\zeta} \right)I_{sign}(\boldsymbol{\zeta,}\boldsymbol{\xi}_{\left( i \right)}\boldsymbol{)}d\boldsymbol{\zeta}$ | (2) |

**Supplementary Movies**

**Supplementary Movie 1. Circular 2D bubble array wet-to-dry transition**

This movie is made from still shots taken with 30-second interval. It shows the bubble array structural evolution over time. Since the bubble array is placed between two glass slides, the liquid evaporation takes place at the outer edge only. The average vertical liquid film thickness decreases over time. The mass loss and the tendency to reduce film area determine the overall shape of the bubble array.

**Supplementary Movie 1. Elongated 2D bubble array wet-to-dry transition**

This movie is made using the same method as Movie 1. During the bubble deposition, we intended to draw a line of bubbles to achieve a different starting geometry from the circular case. The "line" immediately shrinks back to reduce the overall surface energy before the top glass slide is placed to trap the bubbles.
